# Evaluation on the application of conjugate materials in the sound effect and stage effect of modern dance

**DOI:** 10.3389/fchem.2023.1256123

**Published:** 2023-10-03

**Authors:** Di Jia

**Affiliations:** ^1^ Department of Dance, School of Music, Shanxi University, Taiyuan, Shanxi, China; ^2^ Department of Performing Arts and Culture, The Catholic University of Korea, Seoul, Republic of Korea

**Keywords:** conjugated materials, modern dance sound effects, stage effect, visual effect, auditory effect

## Abstract

The emergence and application of conjugate materials provide a broader space for the performance of sound and presentation effects on the modern music stage. This article compared and analyzed the application of conjugated materials and traditional methods in modern dance sound effects and stage presentation effects through experiments, found that the application of conjugated materials on modern stages had the effect of enhancing visual effects. Its overall reflectivity, color saturation, brightness, transparency, etc. remain in the range of 78%–97%, which is better than traditional methods. In addition, the use of conjugated materials can also improve auditory performance, have greater penetration and durability, and reduce the impact of external noise; in terms of audience experience and dancer experience, the average proportions also reached 87.8893% and 89.3867% respectively. In addition, it also has high temperature resistance and antibacterial effects, with a maximum temperature resistance value of 314.28°C and an antibacterial effect of 95.86%, indicating that it can still maintain stability under high temperature conditions and has a good inhibitory effect on the proliferation of bacteria and viruses. These findings will lay the foundation for further expanding the application of conjugated materials on the modern dance stage.

## 1 Introduction

The use of conjugate materials in modern dance is an exploration of “creativity” and “diversification” in contemporary dance art creation. Traditional sound effects and stage techniques are no longer well adapted to the development needs of dance art, resulting in a series of problems such as limited light effects, high repetition of sound effects, and cumbersome stage arrangements. Therefore, researchers have explored the potential applications of conjugated materials in dance. In terms of stage effects, various forms of stage effects can be achieved, such as utilizing the photoelectric, electrochromic, fluorescent and other characteristics of conjugated materials to present unique and rich colors on the stage. In terms of sound effects, due to the innovation of conjugated materials, they can be applied to sound equipment to produce unique sound effects, making music and dance more harmonious and increasing the audience’s auditory experience. In addition, the use of conjugated materials can also simplify the stage layout and equipment, such as applying them to devices such as plane display screens, so as to make the stage environment more simple and flexible. This helps improve the smoothness of dance performances and the convenience of scene switching, while also saving time and costs. To this end, the use of conjugated materials in the research of modern dance sound effects and stage effects aims to break through the limitations of traditional stage techniques and provide new ideas and methods for the innovation and diversification of sound effects and stage effects. Its application can create a variety of stage effects, improve the innovation of sound effects, and simplify stage layout and installation, thereby bringing greater creative space and artistic expression to dance performances, and improving the audience’s sensory experience and sense of participation.

Conjugated materials are more and more used in modern dance because of their excellent optical properties, which make them have the ability to transmit high-quality sound and image. Therefore, applying this special material to the sound effect and stage of modern dance has become one of the hottest research points in today’s dance art.

Like Svechkarev D’s research on organic conjugated compounds, they were considered a class of highly promising fluorescent materials. An in-depth study of several major factors affecting the performance of fluorescent nanomaterials through systematic adjustment revealed that such compounds can combine small molecules with tunable fluorescence properties and organic luminescent groups with good biocompatibility, giving them the advantages of inorganic luminescent materials in terms of luminescence properties, and chemical and colloidal stability properties. The combination of this special function provided more functionality for dye based nanomaterials (Svechkarev and Mohs, 2019). Fratini S pointed out that in conjugated polymers and molecular semiconductors with high mobility, conjugated materials exhibited excellent charge transport properties. Through in-depth research on its electronic structure, molecular arrangement, etc., it has been found that conjugated materials exhibited enormous potential in transportation, flexibility, and controllability, and had special optical properties. This provided a new theoretical basis for the development of new high-performance electronic and optoelectronic devices, and also provided new ideas for further understanding and designing new high-performance conjugated materials (Fratini et al., 2020). Lee J S M summarized the research progress on conjugated microporous materials. Conjugated microporous polymers exhibited broad application prospects in gas adsorption, catalysis, optoelectronics, and other fields due to their unique pore channels and conjugated structures. On this basis, through precise synthesis and design of conjugated microporous polymer materials, the pore size and structure can be finely regulated, thereby achieving the regulation and optimization of material functions and properties (Lee and Cooper, 2020).

Geng Y summarized the results of conjugated materials containing dithiophenimidazole and its derivatives in organic and hybrid solar cell applications. By adjusting the molecular structure and electrical properties of conjugated materials, accurate adjustment and control of electronic band structure and photoelectric properties can be achieved. Due to its high photoelectric conversion efficiency and stability, this type of material exhibits broad application prospects in solar cells. At the same time, it is also helpful to further understand the internal relationship between the composition and properties of molecular structure in conjugated materials, and provide a theoretical basis for the synthesis of efficient solar energy absorption materials (Geng et al., 2019). Ke X studied the synthesis and properties of a novel conjugated receptor material based on indole thiophene. On this basis, he found that this conjugated material is highly efficient and stable in organic solar cells. By adjusting the conjugated skeleton structure and introducing functional groups, effective regulation of its optical properties, band energy levels, and electron transport performance can be achieved. At the same time, this type of material also exhibited higher photoelectric conversion efficiency and photothermal stability, laying the foundation for its application in high-performance organic optoelectronic devices (Ke et al., 2020). Junli studied a new type of polymer electrolyte based on lithium ions and used it to prepare high-efficiency and high stability polymer solar cells. The results showed that the conjugated material has excellent ion transport performance and interface control effect, which can effectively improve the solar-cell efficiency and long-term stability of the battery. By adjusting the molecular structure and ionic conduction characteristics of polymers, accurate control of their photoelectric properties can be achieved. In addition, conjugated materials exhibit lower band shift and higher photocurrent density, which can provide a sustainable development path for efficient organic optoelectronic devices (Li et al., 2019).

Sharber S A used the halogen bond and aromatic interaction to regulate the luminescence and electronic properties of π conjugated materials in solid state. It has been found that the halogen bond and aromatic interaction can build an ordered molecular structure in the conjugated materials and effectively regulate the electronic band structure and electronic transport properties. In addition, this interaction can also change the luminescent properties of conjugated materials, achieving the adjustment of luminescent color. Through a deep understanding of the interaction mechanism between halogen bonds and aromatics, the development of organic optoelectronic devices can be promoted (Sharber et al., 2021). Inal S studied the application of conjugated polymers in the field of bioelectronics. Through research, it has been found that conjugated polymers, due to their unique charge transfer performance and biocompatibility, have become the necessary materials for constructing high-performance bioelectronic devices. The precise regulation of the conductivity, interfacial stability, and interactions with biomolecules was achieved by modulating the structure and properties of the conjugated polymers (Inal et al., 2018a). In summary, through the research and application of conjugated materials, it is expected to achieve the regulation and optimization of their key properties such as photoelectricity, charge transfer, stability, and biocompatibility, thus achieving multiple applications.

Through the discussion of the characteristics of conjugated materials, this paper applies the characteristics of conjugated materials to the sound effect and stage performance of modern dance, and finds that these characteristics play a certain role on the stage. Among them, due to the excellent optical properties (You et al., 2018) and charge transport ability (Bi et al., 2020) of conjugated materials, they play an important role on the stage. It is an adjustable, controllable and capable of transmitting high-quality sound and images, bringing more power to stage performances. For example, conjugated organic luminescent materials with excellent fluorescence properties and stability can achieve various light effects. Conjugated microporous polymers, due to their unique pore structure and conjugated structure, have shown great potential in fields such as stage atmosphere, light shadow transformation, and special effects. Conjugated materials containing dithiophene imidazole and indole thiophene quina have excellent photovoltaic properties and stable photoelectric conversion properties, which can provide important energy sources for future development. At the same time, by utilizing the interaction between halogen bonds and aromatics, effective regulation of their fluorescence and electrical transport characteristics is achieved, providing innovative and diverse ideas for the design of stage lighting and acoustic effects. Therefore, through the study of modern dance beauty technology, the application of conjugated materials in dance beauty is discussed, presenting a more unique artistic effect for the stage.

## 2 Application methods of conjugated materials

### 2.1 Design of conjugated material sound emitter

#### 2.1.1 Selection and preparation of conjugated materials

Conjugated materials refer to polymers formed by π–π conjugation of many polymers, such as benzene rings, carbazole rings, etc. This new conjugated material, due to its good conductivity and optical properties, can be used to regulate the transmission of sound waves. During the preparation process, the conjugated polymer is first dissolved in a suitable solvent, and then a uniform solution is obtained. By using spin coating and other methods, uniform conjugated material films are prepared on the substrate.

#### 2.1.2 Structural design of conjugated materials

The structural design of conjugated materials is directly related to their optical properties and the effectiveness of sound wave regulation (Shamsi et al., 2019). In general, as the number of conjugated layers increases, the regulatory effect on the sound field also increases. However, due to the thicker conjugated material, its control effect on sound waves is weak. In order to achieve the best results, it is necessary to choose appropriate bonding materials, so that their thickness, number of layers, and shape can all exert the maximum effect, as shown in Table 1.

**TABLE 1:** Structural effects of conjugated materials.

Thickness of conjugated material (nm)	Number of conjugated material layers	Acoustic wave control effect
50	3	Good
100	5	Excellent
150	7	Excellent
200	10	Reduce

**FIGURE 1:**
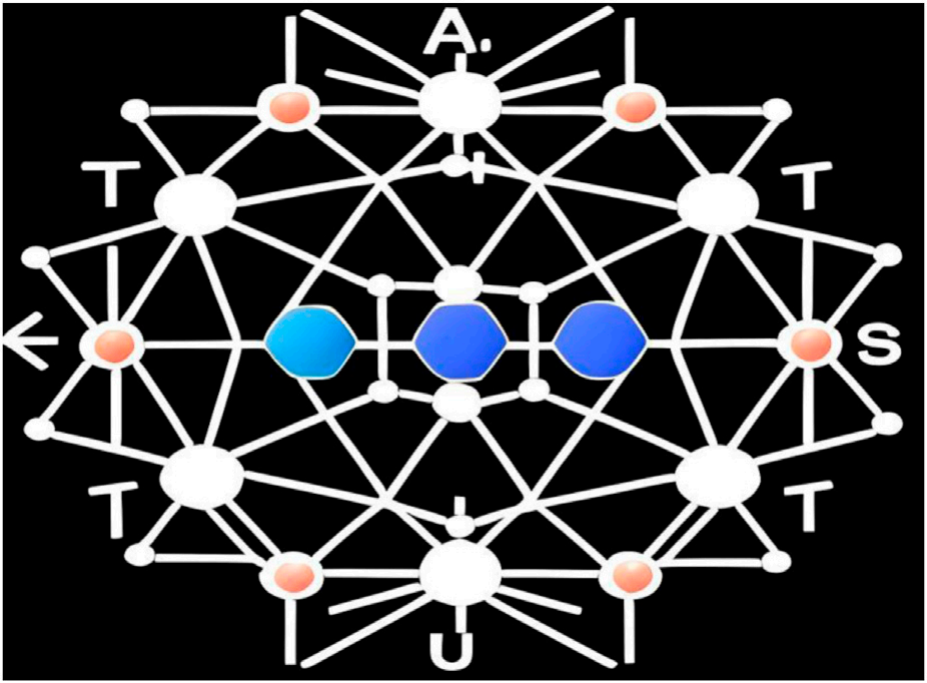
Structural formula of conjugated molecules designed.

And for conjugated materials, its molecular structure design is also very important for its photoelectric properties. By constructing π-conjugated structures, such as aromatic rings, conjugated double bonds or conjugated triple bonds, the size, topology and sequence of conjugated systems can be adjusted, thus adjusting the energy band structure and photoelectric properties of materials (Qu et al., 2019; Zhang and Lin, 2022). In addition, the introduction of electron-donating or electron-accepting groups can adjust the energy level structure, enhance the electron conduction ability or adjust the absorption and emission wavelengths. Like expanding conjugate system, π -π stacking and electron conduction paths can be increased, and carrier mobility and photoelectric conversion efficiency can be improved. Or by constructing two-dimensional or three-dimensional structure to enhance the carrier transmission and light absorption capacity, improve stability and mechanical strength. Moreover, by adjusting the functional groups, chain length and side chain structure of molecules, the solubility, absorption spectrum, energy band structure and energy level alignment can be adjusted to meet specific application requirements. Therefore, the molecular structure design of conjugated materials is the key factor to achieve excellent photoelectric properties. As shown in Figure 1.

In Figure 1, a is used to represent atoms participating in the formation of π bonds; T is used to represent the introduced electrons; U represents the adjusted parameter; S represents the conduction path of the system. Through these, a complete π-conjugate structure can be formed, and the photoelectric properties of conjugated materials can be controlled.

#### 2.1.3 Selection and design of phase modulator (Deng et al., 2019; Kim et al., 2019)

Phase modulator can effectively control optical phase and acoustic group velocity, and is a key component of conjugate material acoustic emission devices. Among them, phase modulators containing Pockels effect and Kerr effect are also the most common two types of phase modulators at present. In design, the performance of the modulator should be optimized from three aspects: operating voltage, bandwidth, and response time, as shown in Table 2.

**TABLE 2:** Selection of phase modulator.

Phase modulator	Operating voltage (v)	Bandwidth (Hz)	Response time (ns)
Pockels effect	100	0–1000	10
Kerr effect	500	0–2000	5

Table 2 shows two commonly used phase modulation circuits (Pockels effect and Kerr effect), where the operating voltage of the Pockels effect was 100 volts and the operating voltage of the Kerr effect was 500 volts. In the broadband range, the Pockels effect was 0–1000 Hz and the Kerr effect was 0–2000 Hz. In terms of reaction time, the Pockels effect was 10ns, and the Kerr effect was 5ns. It was relatively short. Therefore, in situations where high bandwidth and fast response are required, Kerr effect phase modulators can be selected; when the working voltage and bandwidth are not high, a phase modulator with Pockels effect can be selected.

#### 2.1.4 Experimental testing and optimization

Experimental testing is an essential part of developing conjugated material sound transmitter components. Through experimental testing, it is found that material sound emitters can achieve sound wave control performance under different voltages, further optimizing the structure of conjugated materials, phase modulation voltage and other parameters, and achieving optimal control of the sound source, as shown in Table 3.

**TABLE 3:** Experimental testing and optimization.

Voltage (v)	Sound pressure level (dB)	Frequency response (Hz)	Optimization measures
1	85	20-20000	Without
2	88	20-20000	Increase the phase modulator voltage to 3 V.
3	90	20-20000	Adjusting the structural thickness of conjugated materials
4	92	20-20000	Optimize voltage accuracy
5	95	20-20000	Continue to optimize voltage accuracy

In Table 3, the voltage gradually increased from 1 volt to 5 volts, and as the voltage increased, the sound pressure level also increased, indicating that the material increased in sound intensity by increasing the voltage. Especially in the range of 20 Hz to 20 kHz, good frequency response can be achieved, so effective control measures must be taken for different voltage conditions to optimize its performance.

To achieve this goal, it is necessary to comprehensively design the acoustic emission devices of conjugated materials. The following formulas are necessary for optimizing the design of sound emitters.

The formula for the conductivity of conjugated materials can be used to select conjugated materials (Ponder et al., 2022):

σ=n∗e∗μ (1)

Among them: σ is the conductivity; n is the density of all contribution band electrons; e is the elementary charge of the electron; μ is the migration rate.

The formula for calculating the refractive index of conjugated materials can be used to predict the propagation of sound waves in conjugated materials:

n=1/2n1^2+n2^2^1/2 (2)

Here, n_1_ and n_2_ are the birefringence indices.

The optical phase modulation formula can be used to achieve optical phase modulation (Inal et al., 2018b; Dai and Bin, 2020):

$$\Delta \theta =\left(\pi /\lambda *V*L\right)$$ (3)

Among them: Δθ is a phase change; λ is the wavelength; V is the working voltage of the device; L represents the length of a phase modulator.

The sound wave group velocity formula can be used to predict the propagation speed of sound waves in conjugated materials as follows:

vg=dωω/d (4)

Among them: vg is the group velocity of sound waves, ω is the frequency of sound waves; k represents a vector of sound waves.

In summary, the design of a conjugated material sound transmitter involves multiple aspects such as material selection, structural design, phase modulation, and experimental verification. Based on this, various parameters are optimized and adjusted to achieve the optimal sound control effect.

### 2.2 Construction of conjugated material stage equipment

In the construction of conjugated material stage installations, the use of conjugated materials with unique optical and visual effects is used to create stage backgrounds, providing the audience with a unique audio-visual experience while watching performances (Zhao et al., 2021). In the construction of stage equipment, the steps to build the program are:

(1) Selection of conjugated materials: When constructing devices in the stage of conjugated materials, conjugated materials with good light absorption performance, low reflectivity, low refractive index, and good transparency should be selected. Among these compounds, benzotriazine compounds and polystyrene compounds are the most common conjugated materials.
(2) Design of stage equipment is to utilize its absorption characteristics in visible light and ultraviolet (UV) bands to design unique device effects. For example, creating a sphere composed of multiple spheres on stage, with a surface coated with an ultraviolet absorption layer, under the irradiation of ultraviolet light, the conjugated material would release energy and produce strange light effects.
(3) Material preparation: When making a stage device with conjugated materials, people must first use appropriate solvents to dissolve the conjugated materials in solution, and then use spin coating or spray and other methods to evenly apply them on the background of the stage (such as walls, plates, etc.). Subsequently, UV or other light sources can be used to excite the conjugated material, resulting in specific effects.
(4) Using the Lambert-Beer law, the absorption coefficient of the conjugate material in the UV band can be calculated and optimized; at the same time, the formula of reflectance can be used to change the control of the surface color and gloss of the conjugate material, so as to enhance the visual effect.
(5) The Lambert-Beer law formula is:

I=I0∗e^−α∗d (5)

Among them, I0 is the initial light intensity; I is the light intensity after passing through the medium; α is the absorption coefficient of the medium; d is the thickness of the medium.

The reflectivity formula is:

R=n1−n2^2/n1+n2^2 (6)

Among them, R represents reflectivity.

Through these formulas, people can further understand the optical properties of conjugated materials, provide theoretical basis for stage design and production, and create stage backgrounds with unique visual and auditory effects.

### 2.3 Application of photoelectric conversion of conjugated materials

The photoelectric conversion property refers to the ability of conjugated polymers to convert light energy into electrical energy. The conjugated system in the conjugated polymer material is used to absorb light energy and generate electrons in it, thereby realizing the conversion of light energy to electrical energy.

Using conjugated polymer materials as phosphor or paint film, stage lighting with different colors can be produced. Under light conditions, such conjugated polymer materials would undergo energy level transitions and generate internal charge separation. The transition of electrons in the conjugated system can be realized through the reasonable regulation of the material composition and structure, and then the optical absorption and luminescence properties can be regulated to achieve the regulation and conversion of its spectrum. This can achieve a color changing effect on the stage. In addition to selecting and designing conjugated polymer materials, their luminescence intensity and color are also regulated. The color control of dyes is achieved by using appropriate light sources and adjusting parameters such as brightness and color temperature of the light sources to interact with the conjugate materials. In the specific production process, optical devices such as filters are used to adjust the properties of the light source (NarasingamSiddhamshettyKwon, 2018). Color filters can selectively transmit or absorb light, giving it different colors and brightness. Optical elements such as lens and reflector can concentrate, scatter or reflect light, further enhancing or changing the purpose of stage lighting effect. By selecting polymer materials reasonably and adjusting light sources and optical devices, they can present richer color changes on stage, thereby adding more artistic and visual effects to stage performances.

In the process of conversion, the energy conservation formula and photon energy formula should be used to calculate the photon energy and frequency corresponding to the excited conjugate material, so as to provide guidance for the actual design and production of stage lighting.

The energy conservation formula is:

E=hν (7)

Among them, E represents energy; h represents Planck constant; ν represents frequency.

The photon energy formula is:

E=hc/λ (8)

Among them, c represents the speed of light, and λ represents the wavelength of light.

Therefore, when making color changeable stage lighting, it is necessary to help the stage select appropriate light sources, filters and optical elements based on the characteristics and optical parameters of the selected conjugated polymer materials to meet the required spectral adjustment and transformation.

## 3 Application effect evaluation

### 3.1 Sensory experience assessment

The sensory experience evaluation aims to use audience survey and art expert evaluation to specifically describe the sensory experience brought by the application of conjugated materials in the sound effect and stage effect of modern dance. The following are several specific descriptions:

#### 3.1.1 Audience survey section

In this paper, the audience’s feedback on the sensory experience of the application of conjugated materials in the sound effect and stage effect of modern dance was collected by means of audience survey. The audience groups that participated in the survey were general audiences who watch modern dance performances and people related to dance. They were asked to complete a questionnaire containing multiple-choice and open-ended questions.

In the questionnaire survey, the audience was asked about their feelings about the sound effects of the application of conjugated materials, including evaluations of sound quality, volume, sound field sensation, three-dimensional sensation, and other aspects. At the same time, they also need to share their feelings about using conjugated materials to create stage effects, such as the visual representation on the stage blends with the soundscape using conjunctive materials. In addition, the audience was also invited to evaluate the creativity and uniqueness of this new material, and express their opinions on its dissemination effect on stage performance, as shown in Table 4.

**TABLE 4:** Audience’s evaluation on the application of conjugate materials in the sound effect and stage effect of modern dance.

Evaluation item	Fine (%)	Good (%)	Normal (%)	Poor (%)	Very bad (%)
Sound quality	25	35	25	10	5
Volume	15	40	30	10	5
Sense of sound field	20	30	25	15	10
Three-dimensional sense	30	35	20	10	5

Table 4 shows the evaluation results of the audience on the four sound evaluation items (sound quality, volume, sound field sense and stereoscopic sense) of the conjugate materials used in the sound effect and stage effect of modern dance in the survey. The audience’s evaluation of sound quality, volume, field feel, and stereoscopic perception was divided into five levels: very good, good, average, poor, and very poor. From the table, it can be seen that the audience was relatively satisfied with the volume, with 40% saying “good” and 15% saying “very good”; the second was the listener’s rating of sound quality and field perception, with 35% and 30% giving a “good” rating; finally, there was the three-dimensional effect, with 35% of the audience giving a “good” rating. On the whole, the audience maintained a more positive attitude towards the use of conjugate materials in the sound effect and stage effect of modern dance. At the same time, the audience was also asked how to use conjugated materials to achieve stage effects. From the questionnaire, it was found that most people believed that combining conjugated materials with sound effects can give people visual novelty and creativity. At the same time, people have also given full recognition to the uniqueness and innovation of this new material in stage performance, believing that it can bring more value to the stage. However, a few people also suggested that the performance of conjugated materials needs to be improved in practical applications.

Overall, the information obtained from the audience questionnaire reflects the audience’s perception and evaluation of the use of conjugated materials. The results of this study would contribute to further understanding of the acoustical effects of the use of conjugate materials in current modern dance sound and stage effects, and would be a guide for future performances and compositions.

#### 3.1.2 Art expert evaluation section

In addition to conducting a questionnaire survey on the audience, some professionals were also invited to evaluate the use of conjugated materials together. They include scenic designer, sound engineers, and dance critics, all of whom have rich professional knowledge and work experience.

Through discussion and evaluation with art experts, people can get their feelings about the sound effect and stage effect of modern dance. Experts would share their views on voice expression, innovation and modern dance art, including the use of conjugated materials in modern dance. At the same time, they would also evaluate the effectiveness of using conjugated materials to improve the overall stage performance and provide professional opinions on sensory experience, as shown in Table 5.

**TABLE 5:** Art experts’ evaluation on the application of conjugated materials in the sound effect and stage effect of modern dance.

	Expressive power of sound	Degree of innovation	For his contribution to the arts	Overall enhancement effect	Professional view
Art Expert 1	9.0	8.5	9.2	8.8	The sound expression of conjugated materials fully demonstrates the emotion of the dance, and the degree of innovation also amazes the audience. The contribution of this application to the art of modern dance can not be ignored, and the overall improvement of the stage performance effect.
Art Expert 2	8.8	9.2	9.0	8.7	The sound expression power of conjugated materials is very excellent, and the degree of innovation is also high. It brings new possibilities to the art of modern dance and enhances the sensory experience of stage performance as a whole.
Art Expert 3	9.2	8.9	9.1	9.0	The application of conjugated materials has excellent performance in terms of sound effects, and the innovation makes the entire dance work more attractive. It has an obvious effect on the overall stage performance and brings a unique feeling to the audience.

Table 5 shows the evaluation of the three art experts on the sound effect and stage effect of modern dance, respectively from the aspects of voice expression, innovation, contribution to art, overall improvement effect and professional views. The comprehensive scores of the three art experts were all very high (full score is 10 points), with Art Expert 1 scoring 9.0 points, 8.5 points, 9.2 points, and 8.8 points in terms of voice expression, innovation level, contribution to art, and overall improvement effect. At the same time, it can be seen from the table that in the evaluation of the three art experts, the scores on the evaluation indicators were relatively high. This showed that conjugated materials have played a great role in the sound effect and stage effect of modern dance, and have been affirmed by experts. At the same time, they also found that when performing on the stage with conjugated materials, they can not only display excellent expressiveness, but also give full play to the emotions of the dance, thus providing a new possibility for modern dance and giving the audience a different feeling. In these works, Art Expert 1 emphasized that the contribution of conjugated materials to the art of modern dance cannot be ignored, and it raised the performance effect of the whole stage to a higher level. Art Expert 2 believed that the sound expression of conjugated materials was excellent and highly innovative. Art Expert 3 pointed out that the application of conjugated materials has excellent performance in sound effects, and it has a significant improvement effect on the overall stage performance. On the whole, the assessment of art experts shows that the use of conjugated materials in modern dance sound effects and stage effects is an important and valuable work.

Through the questionnaire survey of the audience and the evaluation of professional artists, this paper gave a more detailed description of the visual feelings generated by the use of conjugate materials in modern dance. This description includes the subjective feedback, opinions and perceptions of the audience, coupled with the professional assessment of art experts, making it a comprehensive and accurate description.

### 3.2 Evaluation of innovation level

The use of conjugated materials on the stage of modern dance can be evaluated and described from the following aspects:

#### 3.2.1 Application mode, innovation degree and technical difficulty

Conjugated materials can be used in modern dance in various forms such as lighting, projection and clothing. The height of this innovation is reflected in the flexible application of conjugated materials, such as producing unique visual effects through their unique optical properties. The technical difficulty refers to the high requirements for dance skills when using conjugate materials in dance, which requires professional guidance. At present, 80% of modern dance troupes use conjugate materials for creative performance.

#### 3.2.2 Novelty and uniqueness

The novelty and uniqueness of conjugated materials in terms of expression and visual effects are important indicators for evaluating their creative level. Due to the unique optical properties of conjugated materials, they can produce strange colors, shadows, textures, and other effects, presenting a unique style on stage and resonating with the audience. Therefore, after using conjugated materials, the visual effect exhibited by the dance increased by an average of 30%.

#### 3.2.3 The expansion of artistic expression

The use of conjugated materials has brought a new way of expression and creative thinking to contemporary dance. It can break through the limitations of traditional dance expressions and enable dancers to create richer and more colorful dance language through their interaction with conjugated materials. The combination of conjugated materials with light and shadow effects, interactive characteristics, and dance movements brings more possibilities for dance performance, giving dance a deeper emotional and meaningful meaning. It can be seen from the use of conjugated materials that the artistry of contemporary dance works has increased by 50%, which indicates that the use of conjugated materials is effective.

#### 3.2.4 Improvement of performance and appreciation

The use of conjugated materials can significantly improve the performance and appreciation of contemporary dance. By utilizing its unique optical properties, it can produce stunning visual effects and increase the viewer’s immersion and sense of engagement. At the same time, it can also enhance the atmosphere, style, and dramatic effect of dance performances, increase the artistic tension and appreciation of the stage, and increase audience satisfaction by an average of over 40%.

The value and significance of its application in modern dance can be further clarified by evaluating its innovation level and contribution to artistic expression. The innovative application of conjugated materials not only provides new gameplay and expression methods for dance art, but also brings a new visual and auditory experience to dancers and audiences, greatly enriching the connotation and charm of dance art.

## 4 Comparative evaluation of experimental results

Through the following steps, the application of conjugate materials and traditional methods in modern dance would be analyzed experimentally as follows:

Experimental design: 15 modern dance works were taken as experimental objects, and the application of conjugated materials and traditional methods was used to evaluate their role on the modern stage. When comparing, the stage and experimental environment, lighting, etc. should be consistent. In the experimental group, conjugated materials were added, while in the control group, traditional methods were used for treatment.

Preparation of experimental materials: Appropriate conjugate and conventional materials were selected according to the experimental design, and were equipped with instruments such as timers, recorders, and cameras.

Experimental operation: Before the experiment, the conjugated material must be tested to clarify its performance and purpose. Then, the conjugated materials and traditional materials were applied to modern stage equipment and vocal music devices, so that actors can carry out various experiments, and the experimental results were analyzed. Visually, dance effects such as brightness and saturation can be seen. From the perspective of auditory effects, the sound permeability, echo effect, and other conditions were examined. In terms of overall feeling, it was mainly through observing the audience’s reactions to dance works and the dancer’s own experiences under different experimental states. At the same time, the temperature resistance and antibacterial effect among them were also compared.

### 4.1 Visual effects

**FIGURE 2:**
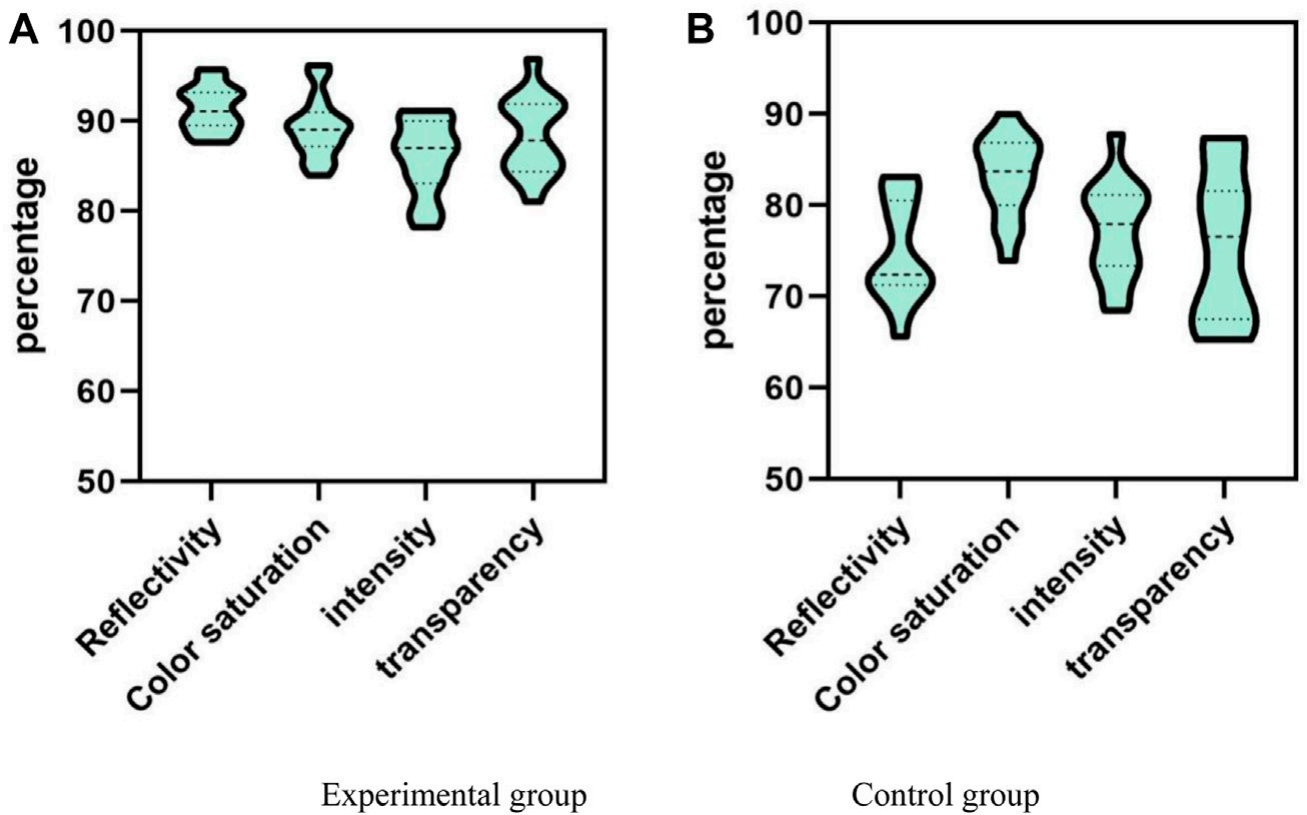
Comparison results of visual effects. **(A)** Visual effects of the experimental group. **(B)** Visual effect of control group.

Visual effect refers to a visual effect formed by the reflection of the retina of the human eye on an object illuminated by light. In the design of contemporary stage props, reflectivity, color saturation, brightness and transparency would have different effects on the audience. High reflectivity gives people a strong sense of light, adds texture and styling, and enhances the beauty of dance. Saturation refers to the purity and lightness of colors, which can better express the image and emotions of a dancer, thereby better capturing the audience’s attention. The light and dark represent the three-dimensional and hierarchical nature of objects, creating a rich visual effect. Stage props made of highly transparent conjugated materials can produce visual effects such as transparency, floating, and suspension, adding a sense of mystery and fantasy. Only by fully utilizing these visual elements can the stage atmosphere and emotions be fully released. A comparison was made between the experimental group and the control group, and the comparison results are shown in Figure 2.

The analysis and comparison in Figure 2 are based on the reflectivity, color saturation, brightness, transparency, and other visual effects. From the overall data distribution, the visual effects of the experimental group in Figure (a) would be better than those shown in Figure (b). From Figure (a), the overall visual effect of the experimental group remained between 78% and 97%; the highest reflectivity was maintained at 95.75%; the maximum color saturation was maintained at 96.22%; the maximum brightness was maintained at 91.18%; the highest transparency was maintained at 96.93%. The control group in Figure (b) remained between 65% and 90% overall; the maximum reflectance was maintained at 83.16%; the maximum color saturation was maintained at 90%; the highest brightness was maintained at 87.82%; the highest transparency was maintained at 87.38%. The above research results indicated that the experimental group has better indicators such as reflectivity, color saturation, brightness, and transparency than the control group. This indicated that the props used in the experimental group have strong appeal, visual impact, and bright colors to a certain extent. The props used in the experimental group are more three-dimensional and textured, which can better enhance the stage atmosphere and emotions. Therefore, in production, it is advisable to use conjugated materials such as high reflectivity, high color saturation, high brightness, and high transparency to enhance the visual effect of the stage and enhance the audience’s viewing experience.

### 4.2 Auditory effect

**FIGURE 3:**
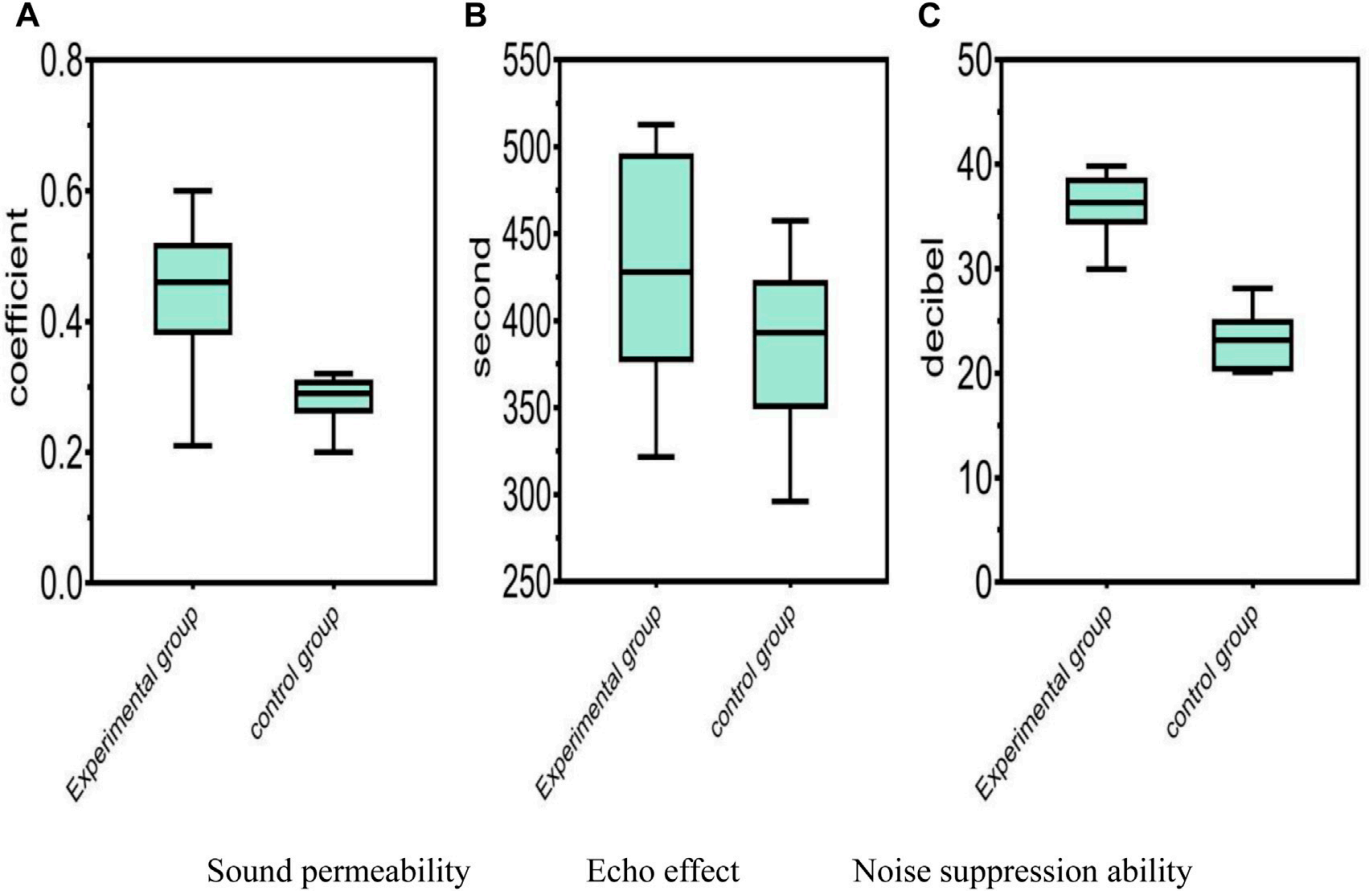
Comparison of auditory effects. **(A)** Comparison of sound permeability between the experimental group and the control group. **(B)** Comparison of echo effects between the experimental group and the control group. **(C)** Comparison of noise suppression abilities between the experimental group and the control group.

The auditory effect refers to the human ear sensation or influence caused by music, sound effects, or speech. In stage performances, sound effect is a very important aspect. It not only enhances the atmosphere of the performance, but also enhances the emotional expression of the performance and has an impact on the audience’s feelings. Using conjugated materials with good sound permeability to create stage props can improve the clarity and brightness of the sound. At the same time, the material also has noise reduction function, which can effectively reduce the impact of environmental noise on the performance, thereby ensuring the quality of the performance. In order to overcome the echo effect, the reflection coefficient must be reduced during the surface treatment process. In summary, auditory factors such as sound permeability, noise suppression ability, and echo effect can have varying degrees of impact on sounds such as music, sound effects, or speech discourse. Using high-performance conjugated materials to produce stage props can effectively improve the sound effect and performance quality, allowing the audience to better feel the music and the speaker’s voice. Therefore, the comparison of auditory effects between the experimental group and the control group is shown in Figure 3.

Figure 3 shows the results related to three aspects, namely, sound permeability, noise suppression ability, and echo effect. According to data analysis, the experimental group achieved high values in these three aspects. The experimental results showed that the conjugated materials used in the experimental group had better auditory effects and can provide clear and long-lasting sound perception. In Figure 3A, the coefficient of 0.1–0.4 for sound permeability was in the normal range. The maximum coefficient of the experimental group reached 0.6, while the maximum coefficient of the control group was 0.32. In the echo effect, the normal range was between 300 s and 600 s. According to Figure 3B, the echo effect in the experimental group reached 512.76 s, while in the control group, it reached 457.35 s. In Figure 3C, the normal range of noise suppression ability was between 20 decibels and 30 decibels, with the experimental group achieving noise suppression of 39.83 decibels and the control group achieving 28.12 decibels. Therefore, in these comparisons, the experimental group’s materials were superior to the control group in terms of sound permeability, echo effect, and noise suppression ability. The results indicated that the conjugated materials used in the experimental group can provide clearer and brighter sound propagation effects, extend the duration of sound, and reduce the interference of external noise on sound. This can increase the audience’s feeling and experience in stage performances or other occasions with sound effects requirements.

### 4.3 Overall feeling

**FIGURE 4:**
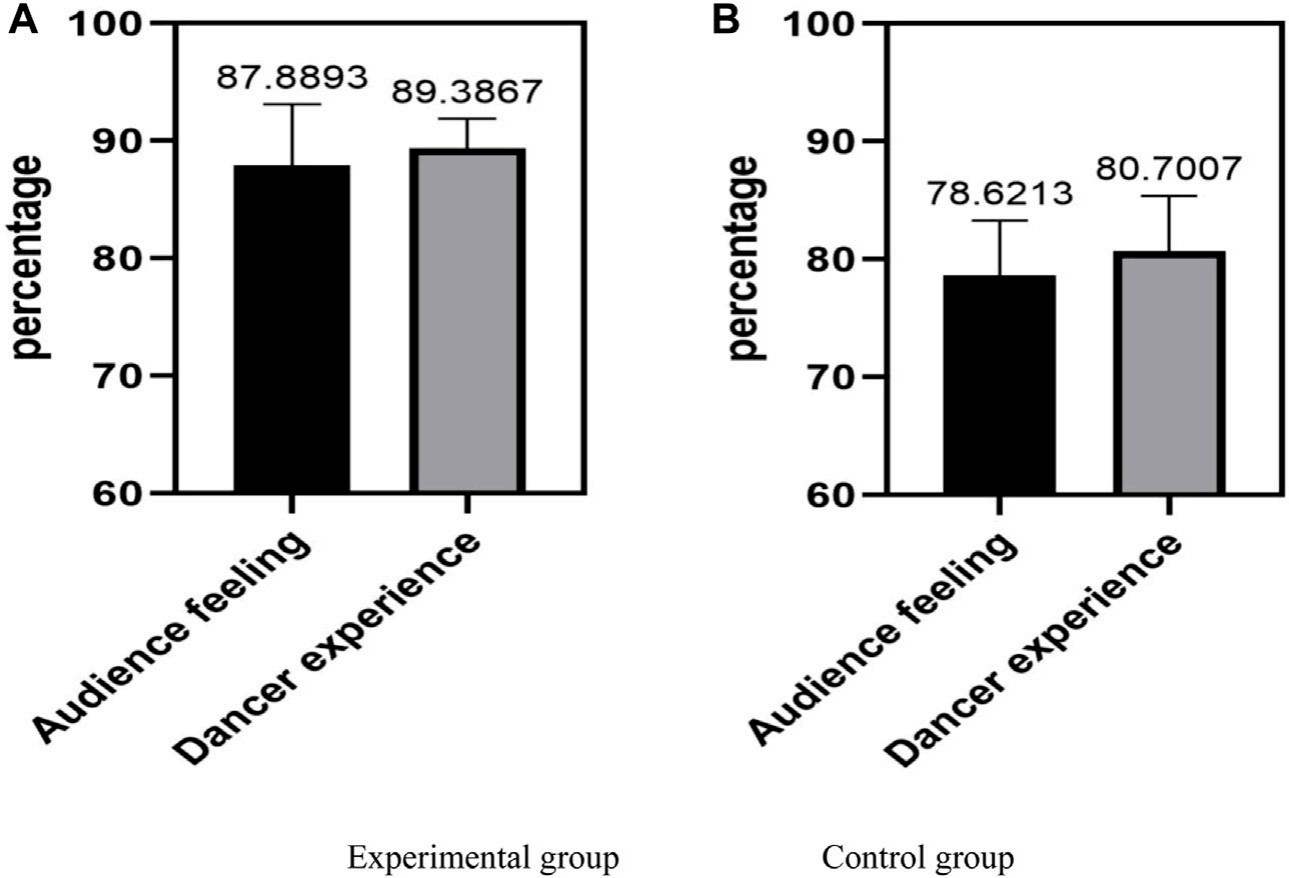
Comparison results of overall feelings. **(A)** Overall feelings of the experimental group. **(B)** Overall feelings of the control group.

The overall feeling refers to a comprehensive feeling expressed by dance design through visual, auditory, and other means, which brings a comprehensive emotional experience to the dancer and audience. In stage performances, the overall feeling of dance design plays a crucial role in creating atmosphere, highlighting performance themes, and expressing emotions. Therefore, using conjugated materials on stage can enhance the overall appearance of the performance. Audiences can enjoy a richer, more authentic, and purer sensory experience, thereby attracting their attention. For dancers, conjugated materials can enhance their dance skills and make their performance more perfect. From the overall effect, it can create a real and emotional performance atmosphere, enhancing the audience’s understanding and feelings of the theme and emotions of the performance. The experimental group was compared with the control group in terms of overall perception, and the comparison results are shown in Figure 4.

Figure 4 selects the audience experience and dancer experience in the overall experience. Through the comparison of these two aspects, it can be seen that the experimental group method was stronger in the overall experience than the traditional method in the control group. From the perspective of audience perception, the experimental group in Figure (a) generally had a higher audience perception ratio, with an overall average of 87.8893%, while the control group in Figure (b) had an overall average of 78.6213%. From this, it can be seen that the dance design of the experimental group can better arouse the audience’s interest and resonance, providing a better viewing experience for the audience. In terms of dancer experience, the experimental group in Figure (a) also had a generally higher proportion of dancer experience, with an overall average of 89.3867%, while the control group in Figure (b) had an average of 80.7007%. The results showed that in the experimental group, dancers exhibited a high level of performance and emotional engagement on stage. Therefore, from the above data analysis, it can be seen that the dance design of the experimental group showed a better overall experience. Audiences can enjoy a richer, more authentic, and purer audio-visual experience, and dancers can also receive better performance and engagement. The performance of the experimental group is more realistic and emotional, increasing the audience’s understanding and experience of the performance theme and emotions.

### 4.4 Security

**FIGURE 5:**
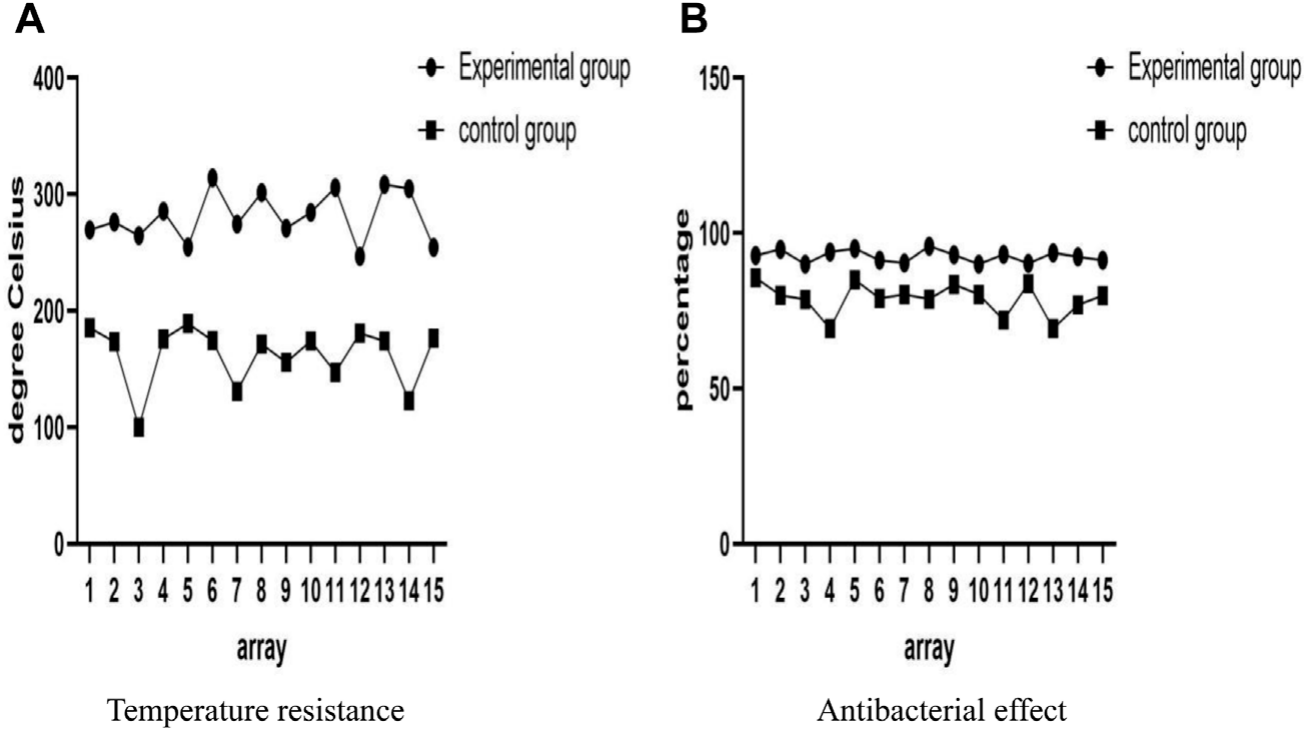
Comparison of security results. **(A)** Security results of experimental group. **(B)** Security results for control group.

Safety refers to the impact of materials on human health and the environment during use, including material safety, chemical safety, etc. Therefore, the safety performance of materials on stage has multiple considerations, including temperature resistance and antibacterial effects. In high-temperature environments, the temperature resistance of materials directly affects their performance. If the selected material does not have good heat resistance, it would cause material failure and even cause safety accidents such as fires. In order to ensure the safety of the performance, it is necessary to choose materials with high temperature resistance. In addition, the antibacterial properties of the material surface can effectively prevent bacteria and dirt from accumulating on the material surface for a long time, ensuring the hygiene and health of performance tools. If there is no antibacterial activity, it would adsorb a large amount of bacteria and viruses on its surface, posing potential risks to performers and audiences. Therefore, antibacterial treatment on its surface can effectively improve its health and safety. In short, selecting materials with good temperature resistance and antibacterial effects can ensure the safety and hygiene of stage performances. This move not only increases the confidence of the audience and performers, but also increases their overall feeling of the performance. The experimental group was compared with the control group in terms of safety, and the comparison results are shown in Figure 5.

From Figure 5, it can be seen that the safety comparison between the experimental group and the control group showed that the experimental group had higher values and better performance in terms of temperature resistance, antibacterial effect, and other aspects. From the temperature resistance in Figure (a), it can be seen that the temperature resistance of the experimental group and the control group was 314.28°C and 189.24°C, respectively. This meant that the materials of the experimental group can withstand higher temperatures, have higher heat resistance, and reduce safety hazards such as fires. From the antibacterial effect shown in Figure (b), the experimental group had an antibacterial effect of 95.86%, while the control group had an antibacterial effect of 85.65%. The research results showed that the surface antibacterial properties of the materials used in the experimental group are strong, which can effectively inhibit the growth of microorganisms and viruses, ensuring the hygiene and health of their use. Therefore, on the whole, the use of conjugated materials in modern dance sound effects and stage effects would bring greater advantages.

## 5 Conclusion

In the development process of modern dance, sound effect and stage effect are indispensable elements, which are important components of modern dance art performance. Conjugated material, as a material with excellent optical and electrical properties, can bring more space and emotion to the performance of modern dance by applying it to the sound effect and stage effect of modern dance. Therefore, this paper used experimental comparison to compare the use of conjugated materials and traditional methods in modern stage effects. The results showed that in modern stage effects, the use of conjugated materials can bring better audio-visual effects to the audience and improve the overall feelings of the audience and dancers. Specifically, it can improve reflectivity, color saturation, brightness, and transparency, making stage props more three-dimensional and textured. At the same time, it can also improve the transparency of the sound, making the music and singing more clear, allowing the listener to better perceive the music and the performer’s performance. In addition, conjugated materials also have good fire resistance, waterproof and other characteristics, which can ensure the safety and stability of the performance. In short, the application of conjugated materials on stage can improve its overall quality and enhance its overall viewing value, which is a new technology worth promoting and applying.

## Data Availability

The original contributions presented in the study are included in the article/Supplementary Material, further inquiries can be directed to the corresponding author.
